# Differentiation of early-stage tumors from benign lesions manifesting as pure ground-glass nodule: a clinical prediction study based on AI-derived quantitative parameters

**DOI:** 10.3389/fonc.2025.1573735

**Published:** 2025-05-19

**Authors:** Shuxiang Chen, Huijuan Zhang, Yifan Chen, Shuo Chen, Wenfu Cao, Yongxiu Tong

**Affiliations:** Department of Radiology, Shengli Clinical Medical College of Fujian Medical University, Fujian Provincial Hospital, Fuzhou University Affiliated Provincial Hospital, Fuzhou, Fujian, China

**Keywords:** lung, pure ground-glass nodule, identification, nomogram, CT, AI, quantitative parameters, benignity

## Abstract

**Objectives:**

Differentiating between benign and malignant pure ground-glass nodule (pGGN) is of great clinical significance. The aim of our study was to evaluate whether AI-derived quantitative parameters could predict benignity versus early-stage tumors manifesting as pGGN.

**Methods:**

A total of 1,538 patients with pGGN detected by chest CT at different campuses of our hospital from May 2013 to December 2023 were retrospectively analyzed. This included CT and clinical data, as well as AI-derived quantitative parameters. All patients were randomly divided into a training group (n=893), an internal validation group (n=382), and an external validation group (n=263). Hazard factors for early-stage tumors were identified using univariate analysis and multivariate logistic regression analysis. Independent risk factors were then screened, and a prediction nomogram was constructed to maximize predictive efficacy and clinical application value. The performance of the nomogram was evaluated using ROC curves and calibration curves, while decision curve analysis (DCA) was used to assess the net benefit prediction threshold.

**Results:**

The final logistic model included nine independent predictors (age, location, minimum CT value, standard deviation, kurtosis, compactness, energy, costopleural distance, and volume) and was developed into a user-friendly nomogram. The AUCs of the ROC curves in the training, internal validation, and external validation cohorts were 0.696 (95% CI: 0.638–0.754), 0.627 (95% CI: 0.533–0.722), and 0.672 (95% CI: 0.543–0.801), respectively. The calibration plot demonstrated a good correlation between observed and predicted values, and the nomogram remained valid in the validation cohort. DCA showed that the model’s predictive performance was acceptable, providing substantial net benefit for clinical application.

**Conclusions:**

The clinical prediction nomogram, based on AI-derived quantitative parameters, visually displays an overall score to differentiate benign lesions from early-stage tumors manifesting as pGGN. This nomogram may serve as a convenient screening tool for clinical use and provides a reference for formulating individualized follow-up and treatment plans for patients with pGGN.

## 1 Introduction

With the increasing use of low-dose spiral CT and the clinical implementation of artificial intelligence (AI)-based auxiliary diagnostic systems (1, 2), an increasing number of asymptomatic ground-glass nodule (GGN) are being detected during routine physical examinations, even in non-smokers. Among these, pure ground-glass nodule (pGGN) have garnered particular attention due to their association with a spectrum of benign and malignant diseases. Benign lesions can be monitored over an extended period, while preinvasive lesions, such as atypical adenomatous hyperplasia (AAH) and adenocarcinoma *in situ* (AIS), can be closely followed to determine the optimal timing for limited resection. In contrast, minimally invasive adenocarcinoma (MIA) and invasive adenocarcinoma (IAC) necessitate immediate surgical intervention. The prevalence of “indolent cancers” presenting as pGGN is on the rise (3–7). The psychological and economic burdens on individuals can significantly affect their quality of life. Overdiagnosis and overtreatment are common risks that further complicate the situation. Therefore, reducing the incidence of these phenomena has become a focal point of screening research (8–10).

The management of pulmonary nodule poses a significant clinical challenge due to their varied nature. Most benign lesions either subside or remain stable over time, often requiring only routine follow-up. Among malignant nodule, there exists a spectrum of neoplastic lesions, ranging from AAH to AIS, MIA, and IA. Early-stage tumors, such as AIS and MIA, have a 5-year survival rate approaching 100% (11), Consequently, for these early-stage lesions, initial observation followed by timely surgical intervention is a reasonable approach. In contrast, IA requires more aggressive management. Thus, accurately distinguishing the nature of persistent pGGN holds significant clinical importance to ensure rational responses from both patients and clinicians. Traditional CT imaging features of benign and malignant lesions often overlap, and the phenomena of “same disease, different imaging signs” and “same sign, different diseases” occur frequently, necessitating further investigation. Achieving an accurate qualitative diagnosis and determining appropriate clinical treatment strategies remain critical challenges for both patients and clinicians. Various factors have been identified as potential predictors for differentiating between benign and malignant lung nodule. These include nodule size, spiculation, lobulation, and other radiological features, as well as patient characteristics such as age, smoking history, and family history of lung cancer. These factors have been incorporated into nomograms to calculate individualized risk scores for GGN nature (3–7). Compared to solid nodule, GGN, particularly pGGN, exhibit relatively indolent behavior. In most cases, patients with pGGN demonstrate favorable survival rates and low recurrence. However, predicting the presence of invasive components within pGGN remains a significant challenge. Conventional CT features of pGGN across different diseases are often similar, and there is a lack of precise quantitative indicators. In recent years, radiomics has made remarkable progress in identifying the degree of invasiveness in pulmonary nodule. However, radiomics requires manual delineation, which is time-consuming, labor-intensive, and prone to inter- or intra-observer variability and manual measurement errors (2, 12–14). As a highly efficient and promising automated method, AI-based quantitative parameters may address these shortcomings. AI can capture subtle differences that are difficult to discern with the naked eye.

With the continuous advancement in the integration of medicine and engineering, AI-assisted diagnostic systems for pulmonary nodule have been widely adopted in clinical practice (5, 6, 15). Leveraging 3D deep convolutional neural networks, AI can accurately capture the complete 3D structural information of pulmonary nodule, which vary in shape and size, and enable the automatic extraction of nodule contours. These systems not only automatically detect the location of pulmonary nodule but also perform rapid quantitative analysis. Various three-dimensional quantitative parameters of pulmonary nodule—such as average CT value, 3D long diameter, maximum area, volume, surface area, compactness, sphericity, and entropy—can reveal subtle changes that are imperceptible to the human eye. These AI systems offer higher sensitivity and repeatability, assisting doctors in enhancing the accuracy of imaging diagnoses, reducing missed diagnoses and misdiagnoses, and minimizing repetitive and labor-intensive tasks (16).

AI is becoming increasingly integrated into all areas of medicine and has gradually demonstrated its advantages in lung cancer screening, segmentation, location identification, classification, and diagnosis of lung nodule in clinical practice (8, 17–20). AI-derived quantitative parameters may provide additional information for differentiating the nature of pGGN, aiding in the early screening, diagnosis, and treatment of malignant pulmonary nodule, while also helping to avoid overdiagnosis and overtreatment of benign lung lesions (9, 21). Therefore, whether AI parameters, which are automatic and convenient, can provide further valuable insights remains an interesting question for further investigation. To the best of our knowledge, no published studies have focused on using AI-derived quantitative parameters to predict the nature of pGGN. In this study, we aim to develop a clinical prediction nomogram based on AI-derived quantitative parameters to differentiate between benign and malignant pGGN. We hope to contribute valuable insights to the field of pGGN management and assist healthcare providers in optimizing patient care and treatment decisions.

## 2 Materials and methods

### 2.1 Patient data

**Figure 1:**
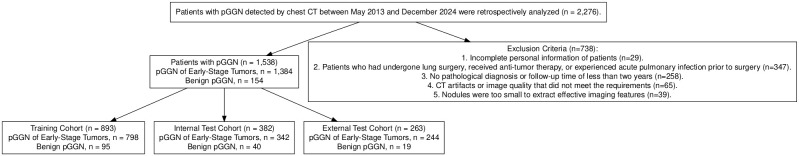
The flow chart showed the enrollment of patients.

Chest CT images and clinical data were retrospectively collected from patients with GGN in different campus of hospital from May 2013 to December 2023, 1538 patients with pGGN were enrolled in the study. including 562 males and 976 females, aged from 26 to 78 years, with an average age of 55 ± 12 years. There were 111 cases of IA, 615 cases of MIA, 48 cases of AIS, 610 cases of AAH and 154 cases of benign diseases. The flow chart showed the enrollment of patients (**Figure 1**).

The type of nodule is often inconsistent even among chest radiologists, for consistency, the AI classification of ground glass nodule was uniformly used as the standard in this study, and then individually verified by two radiologists with more than 5 years of experience, who were blinded to the lesion results, analyzed and recorded the lesion features, including lesion site, number, If there was a disagreement, the decision was made by a third experienced radiologists.

Inclusion Criteria:

1. Complete CT images and clinical records were available for analysis.
2. Patients had not undergone needle biopsy, surgery, radiotherapy, or other related treatments prior to the CT examination.
3. The image format was required to be DICOM.
4. The nodule were identified as pGGN and the size ranged from 3mm to 3cm.
5. For cases with multiple lesions, postoperative pathological results could be correlated with CT images.
6. For pGGN confirmed as malignant by surgical or biopsy pathology, or those that resolved after anti-inflammatory treatment or follow-up, the diagnosis of malignant nodule was based on pathological evidence. If pGGN resolved during follow-up and were considered benign but lacked pathological confirmation, patients were required to undergo follow-up for more than two years.

### 2.2 CT image protocol

All patients were scanned at full inspiration while in the supine position with their hands raised, using either the Somatom Definition AS 128 or the Somatom go. Top scanner. All CT examinations were performed from the apex to the base of the lungs following standard clinical scanning protocols. The tube voltage was set at 120 kV, and automatic tube current modulation was applied. Thin-slice reconstructions were performed with a slice thickness of 1.25 mm. Image post-processing adhered to standardized protocols. Routine chest CT imaging included both pulmonary and mediastinal window settings.

### 2.3 Image analysis

The chest CT images were automatically detected and delineated using the Artificial Intelligence-Assisted Diagnosis System (https://www.shukun.com/product/). This system performed automatic segmentation and extracted quantitative parameters, including mean CT value, maximum CT value, minimum CT value, median CT value, standard deviation, kurtosis, skewness, entropy, compactness, sphericity, energy, surface area, maximum 3D diameter, costopleural distance, mass, and volume. Pathological results, along with the following demographic and clinical data, were also collected, such as sex, age, and lesion location.

### 2.4 Statistical analysis

The dataset collected from one campuses of our hospital was randomly divided into training and validation cohorts at a ratio of 7:3. Cases collected from another hospital campuses served as an external validation set. Continuous variables are presented as median (interquartile range), and categorical variables are expressed as absolute counts and percentages (%). In the univariate analysis, the chi-square test or Fisher’s exact test was used to analyze categorical variables, while the Student’s t-test or rank-sum test was applied for continuous variables. In the training cohort, least absolute shrinkage and selection operator (LASSO) logistic regression analysis was performed for multivariate analysis to screen for independent risk factors. Based on these results, a practical nomogram was developed to differentiate benign from Early-Stage Tumor pGGN. The performance of the nomogram was evaluated using the receiver operating characteristic (ROC) curve, calibration curve, and decision curve analysis (DCA). Statistical significance was defined as a two-sided p value < 0.05. All statistical analyses were conducted using R software (version 4.2.2) and MSTATA software (www.mstata.com).

## 3 Results

### 3.1 Patient characteristics

Univariate analyses were performed to compare indices between different cohort (**Table 1**, **Supplementary Table 1**). The baseline demographic and clinical characteristics of the study population were analyzed across three cohorts: the training cohort, which included 893 individuals, the internal test cohort, comprising 382 participants, and the external test cohort, consisting of 263 subjects. The distribution of sex revealed that 63.9% of participants in the training cohort were female, compared to 61.3% in the internal test cohort and 65.0% in the external test cohort, with no significant differences (p = 0.559). Similarly, there were no significant differences in median age across the cohorts (p = 0.802).

**Table 1:** Patient demographics and baseline characteristics.

Characteristic	Cohort			p-value^2^
	Training Cohort, N = 893^1^	Internal Test Cohort, N = 382^1^	External Test Cohort, N = 263^1^	
Sex				0.559
Female	571 (63.9%)	234 (61.3%)	171 (65.0%)	
Male	322 (36.1%)	148 (38.7%)	92 (35.0%)	
Age				0.802
Median (IQR)	54 (47, 65)	55 (47, 65)	54 (48, 64)	
Location				0.209
LLL	176 (19.7%)	74 (19.4%)	34 (12.9%)	
LUL	302 (33.8%)	113 (29.6%)	96 (36.5%)	
RLL	93 (10.4%)	43 (11.3%)	28 (10.6%)	
RML	53 (5.9%)	32 (8.4%)	19 (7.2%)	
RUL	269 (30.1%)	120 (31.4%)	86 (32.7%)	
Mean_ct_value				0.093
Median (IQR)	-654 (-716, -568)	-643 (-711, -565)	-639 (-698, -547)	
Maximum_ct_value				<0.001
Median (IQR)	-254 (-423, 31)	-220 (-390, -8)	-132 (-311, 137)	
Minimum_ct_value				<0.001
Median (IQR)	-922 (-979, -855)	-926 (-984, -855)	-945 (-1,004, -887)	
Median_ct_value				0.148
Median (IQR)	-668 (-724, -582)	-656 (-718, -565)	-654 (-711, -566)	
Standard_deviation				<0.001
Median (IQR)	124 (97, 159)	127 (101, 159)	138 (112, 173)	
Skewness				0.204
Median (IQR)	0.40 (0.20, 0.80)	0.40 (0.20, 0.80)	0.50 (0.20, 0.90)	
Kurtosis				0.013
Median (IQR)	0.10 (-0.30, 1.00)	0.20 (-0.30, 0.90)	0.40 (-0.20, 1.30)	
Entropy				<0.001
Median (IQR)	4.80 (4.10, 5.60)	4.80 (4.10, 5.70)	5.40 (4.75, 6.30)	
Compactness				0.017
Median (IQR)	0.59 (0.53, 0.63)	0.59 (0.54, 0.64)	0.57 (0.53, 0.62)	
Sphere				0.016
Median (IQR)	0.84 (0.81, 0.86)	0.84 (0.82, 0.86)	0.83 (0.81, 0.85)	
Energy×107				<0.001
Median (IQR)	1.50 (0.91, 3.76)	1.51 (0.83, 3.68)	3.10 (1.60, 5.60)	
Surface				<0.001
Median (IQR)	20 (14, 35)	21 (15, 39)	30 (19, 57)	
Maximum3Ddiameter				<0.001
Median (IQR)	6.0 (5.0, 7.0)	6.0 (5.0, 8.0)	7.0 (5.0, 10.0)	
Costopleura.distance				0.226
Median (IQR)	9 (5, 14)	9 (5, 15)	8 (5, 14)	
Mass				<0.001
Median (IQR)	28 (13, 63)	30 (14, 72)	56 (27, 140)	
Volume				<0.001
Median (IQR)	43 (22, 94)	47 (21, 112)	148 (76, 356)	

^1^n (%).

^2^Pearson’s Chi-squared test; Kruskal-Wallis rank sum test.

Regarding lesion location, the highest proportion of lesions was observed in the left upper lobe (LUL) across all cohorts, followed by the right upper lobe (RUL) and the left lower lobe (LLL), with varying percentages among the cohorts. The mean CT value did not differ significantly between the cohorts (p = 0.093). However, statistically significant differences were observed for several other parameters, including the maximum CT value, minimum CT value, median CT value, standard deviation, skewness, kurtosis, entropy, compactness, sphericity, and energy, with all p-values below 0.001. Additionally, parameters such as surface area, maximum 3D diameter, costopleural distance, mass, and volume also showed significant variations among the cohorts (p-values below 0.001 or 0.017).

### 3.2 LASSO regression and hyperparameter tuning

LASSO was employed to select the most predictive features while addressing multicollinearity and overfitting. LASSO applies an L1 penalty (absolute value of coefficients), which shrinks less important feature coefficients to zero. Features with negligible contributions to the prediction task are thus excluded. For example, Eliminated Features: Sex_Male, Mean_ct_value, Maximum_ct_value, and Median_ct_value had coefficients shrunk to zero (**Table 2**), indicating their minimal discriminatory power in the model.

**Table 2:** The coefficients of Lasso regression analysis.

variable	Coefficient
(Intercept)	2.6715830164
Sex_Male	0.0000000000
Age	-0.0329683761
Location_LUL	0.0000000000
Location_RLL	0.0000000000
Location_RML	0.0000000000
Location_RUL	0.4434778774
Mean_ct_value	0.0000000000
Maximum_ct_value	0.0000000000
Minimum_ct_value	-0.0004195069
Median_ct_value	0.0000000000
standard_deviation	0.0031438836
Kurtosis	-0.0622413126
Skewness	0.0000000000
Entropy	0.0000000000
Compactness	0.4447908252
sphere	0.0000000000
Energy.107	0.0127810360
Surface	0.0000000000
Maximum3Ddiameter	0.0000000000
Costopleura.distance	0.0239581236
Mass	0.0000000000
Volume	-0.0005989829

**Figure 2:**
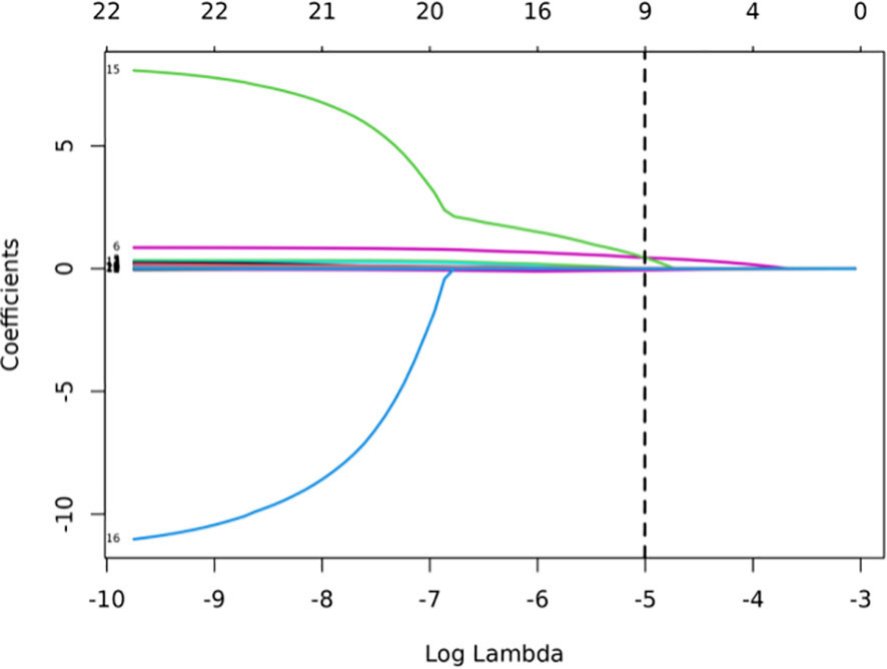
LASSO coefficient profiles of the features. Lasso Regression Coefficient Path Plot (λ = 0.0067).

Retained Features: Age, Location_RUL, and Volume had non-zero coefficients, reflecting their clinical relevance and statistical significance (**Table 2**, **Figure 2**). For redundancy reduction, radiomic features often exhibit high correlation (e.g., Mean_ct_value, Maximum_ct_value, and Median_ct_value). LASSO automatically selects one representative feature from correlated clusters, Only Minimum_ct_value was retained from the CT value family, as it captured unique variance not explained by other correlated features (**Figure 2**).

**Figure 3:**
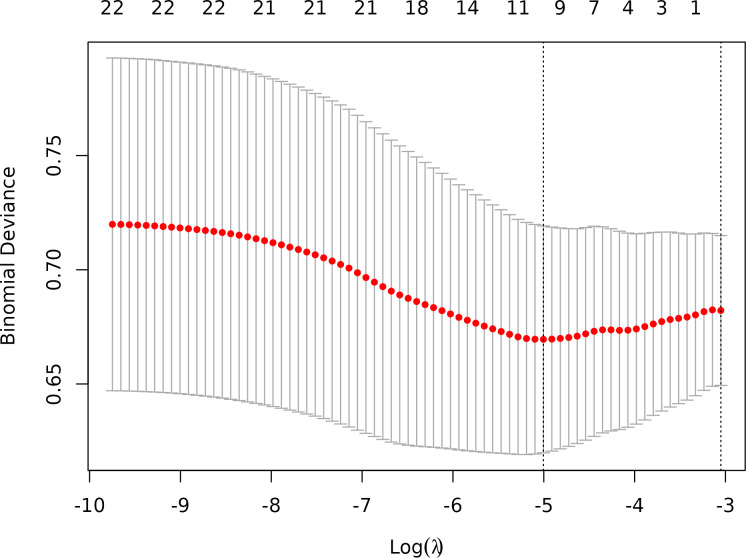
Optimal feature selection of cross-validation. Lasso Regression Cross-Validation Plot.(λ = 0.0067).

**Figure 4:**
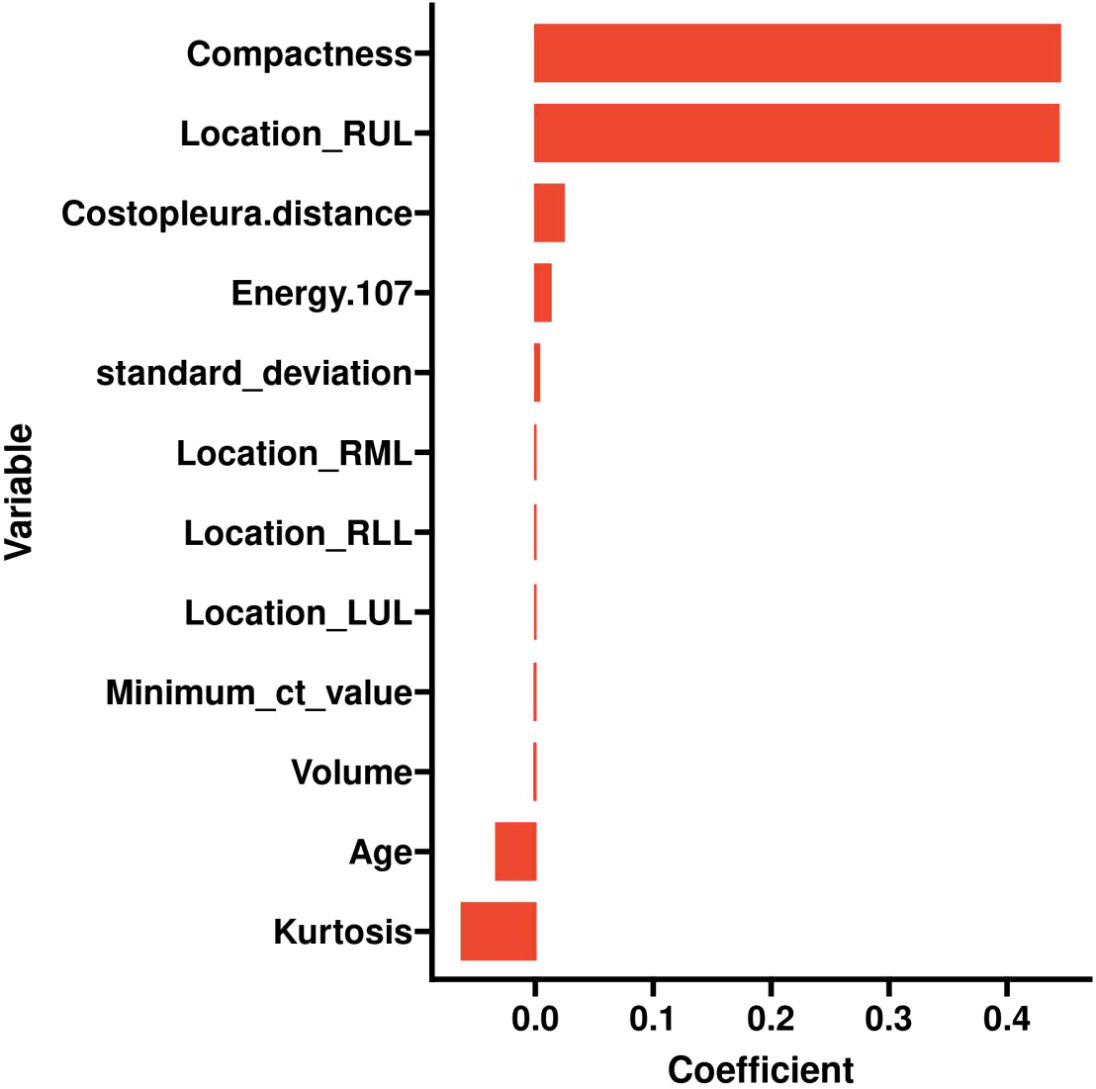
Histogram of the coefficients of the selected feature.

Hyperparameter Optimization, The hyperparameter λ (lambda) controls the strength of the L1 penalty and was optimized as follows:

Ten-fold cross-validation was performed on the training cohort (N=893) to select λ.The “one standard error (1-SE) rule” was applied to choose the most parsimonious model within 1 SE of the minimum mean squared error (MSE). The optimal λ value (0.0067) balanced model complexity and predictive accuracy (**Figure 3**). This λ retained 9 out of 21 features, achieving sparsity without sacrificing discriminative performance (AUC: 0.696 in training; **Table 3**). Finally, the features of AI CT after screening were entered into a support vector machine (SVM) classifier to establish a model that distinguished between benign and malignant pGGN (**Tables 2**, **4**, **Figure 4**). Further multivariate logistic analyses were carried out in different cohorts.

**Table 3:** AUC values and 95% confidence intervals for datasets.

Dataset	AUC Value	AUC 95% Confidence Interval
train_cohort	0.696	(0.638-0.754)
validation_cohort	0.627	(0.533-0.722)
external_cohort	0.672	(0.543-0.801)

AUC calculated using the model predictions; confidence intervals are estimated using DeLong’s method.

**Table 4:** Results of multivariate logistic regression for training cohort.

Characteristic	N	Event N	OR^1^	95% CI^1^	P-value
Age	893	798	0.96	0.94, 0.98	<0.001
Location					
LLL	176	148	—	—	
LUL	302	267	1.50	0.82, 2.73	0.189
RLL	93	84	1.15	0.49, 2.71	0.753
RML	53	48	1.41	0.49, 4.02	0.522
RUL	269	251	2.42	1.24, 4.72	0.010
Minimum_ct_value	893	798	1.00	1.00, 1.00	0.075
standard_deviation	893	798	1.01	1.00, 1.02	0.007
Kurtosis	893	798	0.89	0.78, 1.02	0.093
Compactness	893	798	7.58	0.30, 189.77	0.217
Energy×10^7^	893	798	1.03	1.01, 1.05	0.010
Costopleura.distance	893	798	1.04	1.00, 1.07	0.024
Volume	893	798	1.00	1.00, 1.00	0.001

^1^OR, Odds Ratio; CI, Confidence Interval.

### 3.3 Predictive model

**Figure 5:**
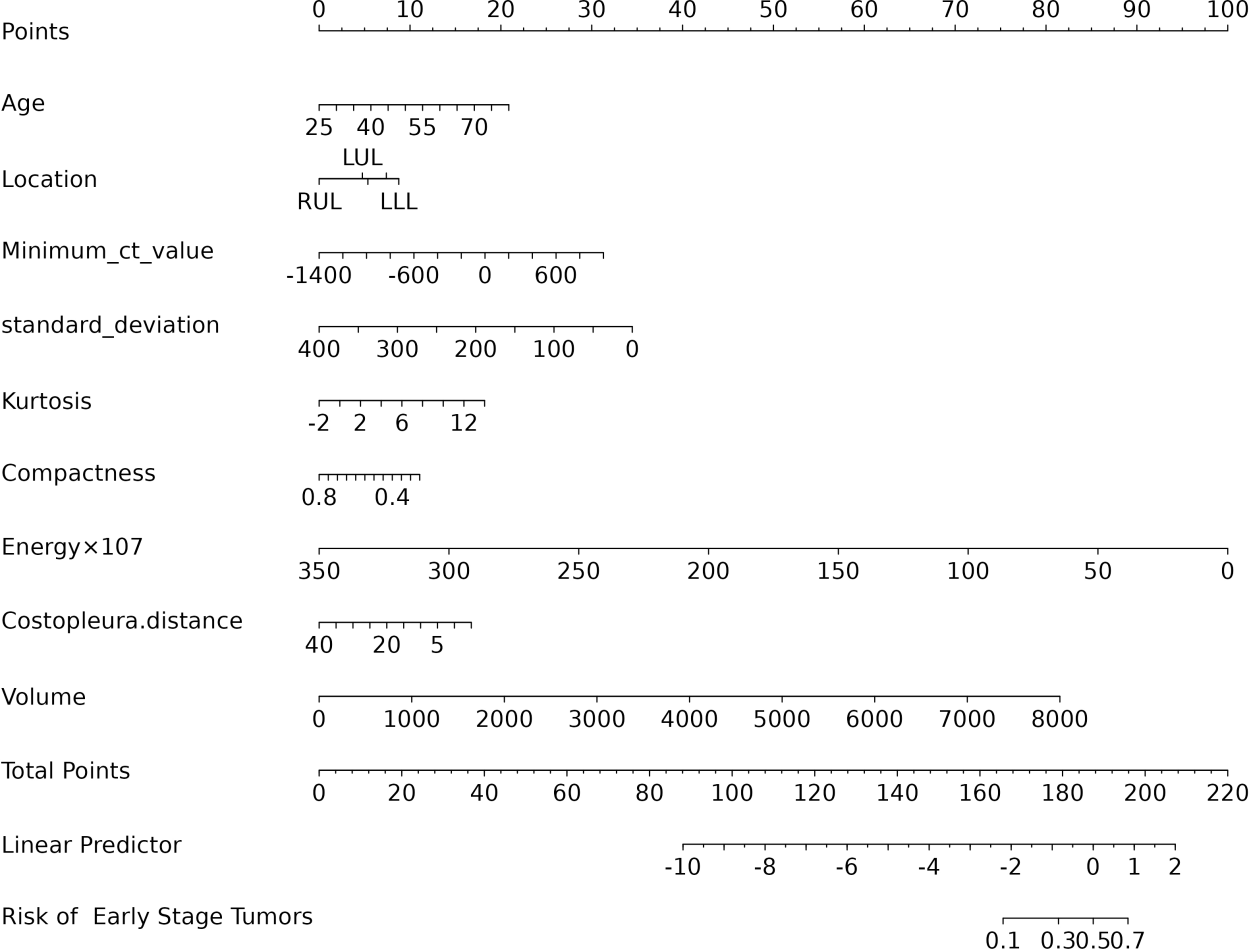
Nomogram of prediction model.

**Figure 6:**
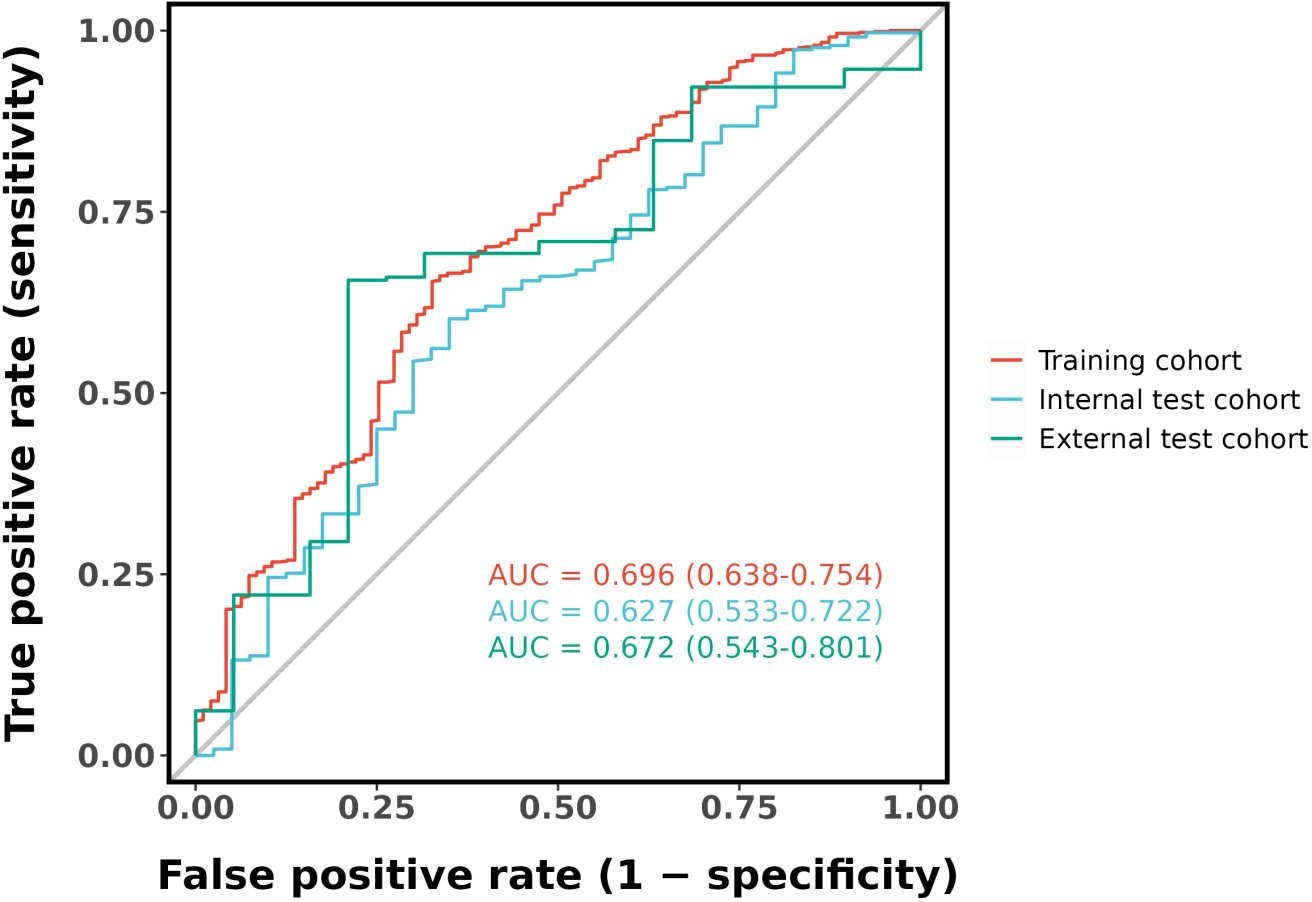
ROC curves of the nomogram.

The final logistic model included 9 independent predictors (Age, Location, Minimum_ct_value, standard_deviation, Kurtosis, Compactness, Energy, Costopleura.distance, and Volume) and developed as a simple-to-use nomogram (**Figure 5**). The AUCs of the model in different cohorts (**Figure 6**, **Table 3**).

**Figure 7:**
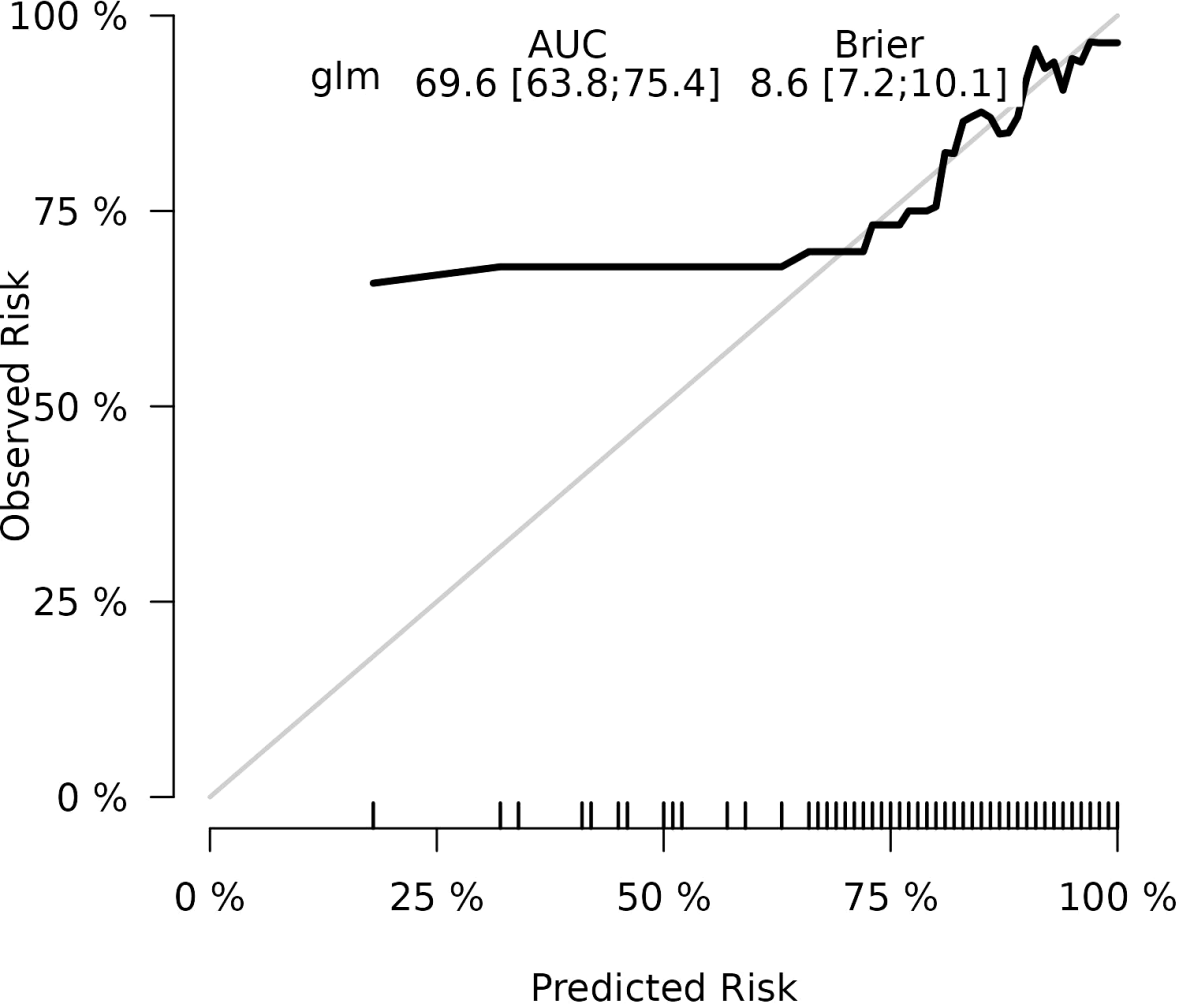
Calibration curve of the nomogram prediction mode for the training cohort.

**Figure 8:**
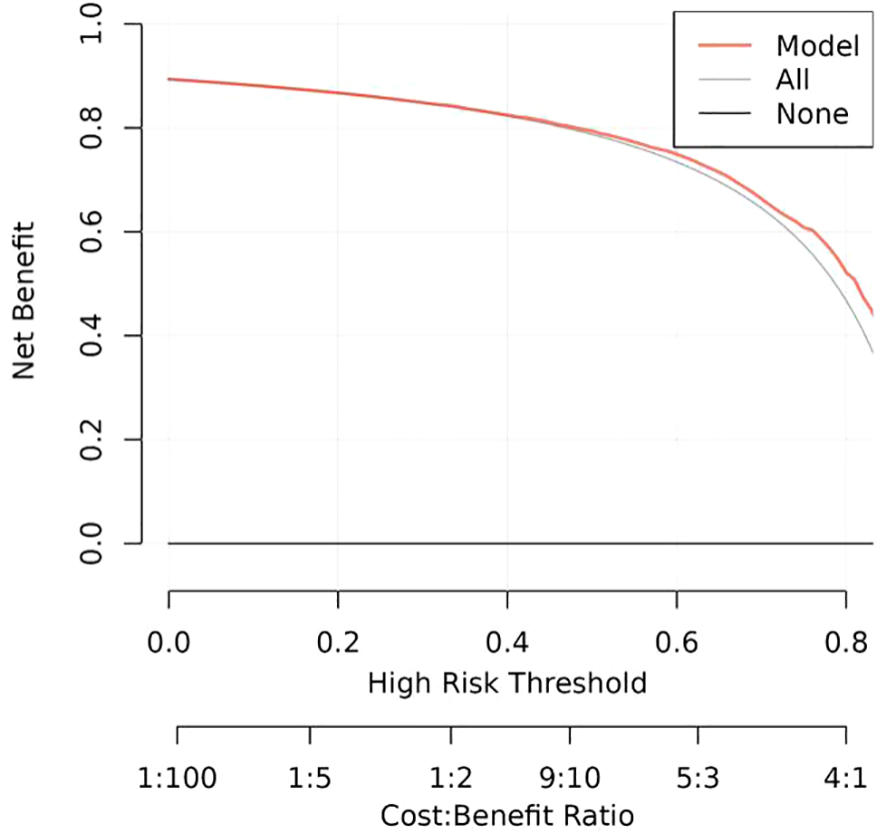
Decision curve analysis of the nomogram of the training cohort.

The calibration plots of the nomogram in different cohorts demonstrate a good correlation between the observed and predicted Status (**Figure 7**). The results showed that the original nomogram was still valid for use in the validation sets, and the calibration curve of this model was relatively close to the ideal curve, which indicates that the predicted results were consistent with the actual findings. A high-risk threshold probability reflects the likelihood of substantial discrepancies in the model’s predictions when clinicians encounter significant challenges while using the nomogram for diagnostic and decision-making purposes. This study demonstrates that the nomogram provides notable net benefits for clinical application, as evidenced by its DCA curve (**Figure 8**).

### 3.4 Clinical implications of misclassification

Detailing FP/FN cases and mitigation strategies (**Table 5**). Training Cohort (N=893),False Positives (FP): 62 cases (6.9%), Primary Causes: Benign inflammatory nodules with high radiomic similarity to malignancies (e.g. high Compactness, OR=7.58, p=0.217).Small nodules (Volume < 30 mm³) misclassified due to partial volume effects. False Negatives (FN): 33 cases (3.7%) Primary Causes: Early-stage tumor with low standard_deviation (p=0.007) or atypical Kurtosis (p=0.093).Subpleural nodules where Costopleura.distance was overestimated. Sensitivity-Specificity Inverse Relationship: At 0.3 threshold, sensitivity improves (92.5%) but at the cost of higher FP (41.7%).At 0.7 threshold, specificity rises (89.7%) but misses 35.9% of true positives. Optimal Threshold Selection: For screening, we recommend 0.3 threshold (minimize missed cases).For diagnostic confirmation, 0.7 threshold reduces unnecessary procedures.

**Table 5:** FP/FN cases and mitigation strategies in different cohort.

Cohort	FP Rate	FN Rate	Key Findings
Training (N=893)	6.9%	3.7%	Early-stage malignancies with low standard_deviation (p=0.007) or atypical Kurtosis (p=0.093).
Internal (N=382)	8.1%	5.2%	Higher FP in nodules with high Energy.107 (p=0.169).
External (N=263)	4.9%	7.2%	FN driven by low Minimum_ct_value (p=0.044).

## 4 Discussion

In this study, we developed and validated a nomogram based on AI-derived quantitative Parameters to differentiate benign lung lesions from early-stage tumors manifesting as pure ground-glass. The primary predictors incorporated into the nomogram included age, lesion location, minimum CT value, standard deviation, kurtosis, compactness, energy, costopleural distance, and volume, all of which were statistically significant in multivariate logistic regression analysis. Similarly, Yang et al. (22) developed a risk prediction model for pGGN invasiveness using meta-analysis-derived features, further validating the utility of radiomics in this context. In our study, we based on AI-derived quantitative Parameters, which rarely reported in the previous literature.

Many literatures have reported that imaging signs of pulmonary nodule, such as lobulation, spiculation, pleural indentation, vacuole sign, and vascular convergence, are suggestive for differentiating malignant lesions (23). However, these signs are less frequently observed in pGGN, and their density is often faint, making it challenging to accurately evaluate the benign or malignant nature of pGGN. Additionally, there is considerable overlap in CT imaging features between benign and malignant lesions. Conventional two-dimensional CT feature analysis has certain limitations, including empirical bias, subjectivity, and insufficient specificity, which cannot be reasonably quantified or accurately assessed. The AI-based quantitative parameters included in this study can help address these limitations.

Our findings are consistent with the existing literature in many respects (22, 24–26), The inclusion of parameters such as energy and compactness in our model reflects their importance in capturing the heterogeneity and morphological complexity of pGGNs, which are often associated with malignancy. For instance, the maximum diameter, regular shape, mean CT value, and lobulation have been previously identified as significant predictors in prior research. However, our study uniquely underscores the importance of quantitative features such as Minimum_ct_value, standard_deviation, kurtosis, compactness energy, costopleura distance, and volume, which are acquired through AI. These features were not emphasized in earlier studies. This discrepancy may be attributed to AI’s ability to extract more quantitative information that is difficult for the human eye to discern.

Asymptomatic pGGN in the lung often persist in clinical practice and represent the most overlapping benign and malignant pulmonary nodule. There is currently no consensus on the management strategy, which often imposes a psychological burden on patients. Clinical attention should focus on early screening, accurate diagnosis, and appropriate treatment of malignant nodule, while also reasonably controlling the frequency of follow-up to avoid overdiagnosis and overtreatment (27, 28).There are ongoing controversies surrounding the management strategy for persistent pGGN. Currently, clinical diagnosis and treatment strategies are primarily based on the dynamic changes observed through manual measurements on routine CT scans, as well as non-quantitative features assessed visually (6, 29–31). While AI-derived metrics offer advantages such as reflecting the natural growth of pGGN and quantitatively capturing subtle changes that are difficult to identify with the naked eye during follow-up, they are also helpful for personalized management.

Our results are consistent with prior research demonstrating the prognostic value of radiomic features in lung nodule assessment. For example, our focus on pGGNs aligns with the growing recognition of their unique clinical behavior, as discussed in studies by Sun et al. (31) and Yang et al. (22). The moderate AUC values in our study are comparable to those reported in similar radiomic models, suggesting that while these tools are promising, they are not yet definitive and should complement, not replace, clinical judgment.

In addition, our study visually displays the overall scores of benign and malignant pGGN patients using a nomogram. This provides clinicians with a quantitative tool to predict benignity or early-stage tumors manifesting as pGGN more accurately than traditional methods, thereby aiding in better risk stratification. Moreover, the early identification of high-risk individuals through this nomogram can facilitate timely interventions, potentially reducing morbidity and mortality.

## 5 Limitations and future directions

Our study has several limitations that should be acknowledged. First, the current predictive accuracy of our models (with AUC values of 0.696, 0.627, and 0.672) leaves room for improvement. These results align with previous studies that have explored radiomic features for pGGN characterization (31). Ensemble learning or deep learning methodologies may could enhance classification performance. Our next steps will systematically evaluate advanced ensemble methods (e.g.Stacking or Boosting);Incorporate lightweight deep learning architectures (e.g.EfficientNet) to balance performance and computational cost and optimize model generalizability through expanded sample sizes. The moderate performance of our model suggests that while radiomic features provide valuable insights, further refinement—such as incorporating advanced machine learning techniques or additional biomarkers—could enhance predictive accuracy. The cohort consisted exclusively of patients from China, which may limit its representativeness of the broader global population. Expanding the sample size through multi-center studies is a crucial direction for future research, as it will be essential to validate the generalizability of our findings. The cases included in this study comprised all cases with pathological results or follow-up absorption, which introduced a degree of selection bias. Future studies should adopt a prospective design and include all suspected cases in advance, regardless of final diagnosis or follow-up, to avoid retrospective bias. In addition, the number of benign cases was relatively limited. Furthermore, there may be unmeasured confounders that were not accounted for in our model. Furthermore, incorporating novel predictors or biomarkers could improve the predictive accuracy of the nomogram, highlighting the need for further investigation.

In summary, our study presents a practical nomogram for predicting malignancy in pGGNs, leveraging clinical and radiomic features. While the model demonstrates moderate performance, its integration into clinical practice could aid in risk stratification and guide personalized follow-up strategies.

## Data Availability

The datasets generated and/or analyzed during the current study are not publicly available due sharing data is not included in our research institution review board but are available from the corresponding author on reasonable request. Requests to access the datasets should be directed to 527336100@qq.com.
