# Corrigendum: Differentiation of early-stage tumors from benign lesions manifesting as pure ground-glass nodule: a clinical prediction study based on AI-derived quantitative parameters

**DOI:** 10.3389/fonc.2025.1631465

**Published:** 2025-06-03

**Authors:** Shuxiang Chen, Huijuan Zhang, Yifan Chen, Shuo Chen, Wenfu Cao, Yongxiu Tong

**Affiliations:** Department of Radiology, Shengli Clinical Medical College of Fujian Medical University, Fujian Provincial Hospital, Fuzhou University Affiliated Provincial Hospital, Fuzhou, Fujian, China

**Keywords:** lung, pure ground-glass nodule, identification, nomogram, CT, AI, quantitative parameters, benignity

In the published article, the **Reference** for 5, 11, 27, 28 was incorrectly written as “5. Luo W. Predicting cervical cancer outcomes: statistics, images, and machine learning. Front Artif Intell. (2021) 4:627369. doi: 10.3389/frai.2021.627369

11. Seyfried F, von Rahden BH, Miras AD, Gasser M, Maeder U, Kunzmann V, et al. Incidence, time course and independent risk factors for metachronous peritoneal carcinomatosis of gastric origin–a longitudinal experience from a prospectively collected database of 1108 patients. BMC Cancer. (2015) 15:73. doi: 10.1186/s12885- 015-1081-8

27. Kitayama J, Ishigami H, Yamaguchi H, Sakuma Y, Horie H, Hosoya Y, et al. Treatment of patients with peritoneal metastases from gastric cancer. Ann Gastroenterol Surg. (2018) 2:116–23. doi: 10.1002/ags3.2018.2.issue-2

28. Hemodynamic Differences in Intracranial Aneurysms before and after Rupture.”

It should be “5. Wan YL, Wu PW, Huang PC, Tsay PK, Pan KT, Trang NN, et al. The use of artificial intelligence in the differentiation of malignant and benign lung nodules on computed tomograms proven by surgical pathology. Cancers (Basel). (2020) 12:2211. doi: 10.3390/cancers12082211.

11. Yotsukura M, Asamura H, Motoi N, Kashima J, Yoshida Y, Nakagawa K, et al. Long-term prognosis of patients with resected adenocarcinoma *in situ* and minimally invasive adenocarcinoma of the lung. J Thorac Oncol. (2021) 16:1312-20. doi: 10.1016/j.jtho.2021.04.007.

27. Ye T, Wu H, Wang S, Li Q, Gu Y, Ma J, et al. Radiologic identification of pathologic tumor invasion in patients with lung adenocarcinoma. JAMA Netw Open. (2023) 6:e2337889. doi: 10.1001/jamanetworkopen.2023.37889.

28. Chang GC, Chiu CH, Yu CJ, Chang YC, Chang YH, Hsu KH, et al. Low-dose CT screening among never-smokers with or without a family history of lung cancer in Taiwan: a prospective cohort study. Lancet Respir Med. (2024) 12:141-52. doi: 10.1016/S2213-2600(23)00338-7.”

The authors apologize for this error and state that this does not change the scientific conclusions of the article in any way. The original article has been updated.

