# Supplementary material for: Differentiation of early-stage tumors from benign lesions manifesting as pure ground-glass nodule: a clinical prediction study based on AI-derived quantitative parameters
Source: Front Oncol. 2025 May 19;15:1573735. doi: 10.3389/fonc.2025.1573735 (PMC12127194; doi:10.3389/fonc.2025.1573735)
Supplement: Supplementary file 1 [file Table1.doc]

**Supplementary**

Table S1 Patient demographics and baseline characteristics

| **Characteristics** | **Training Cohort** | | | **Internal Test Cohort** | | | **External Test Cohort** | | |
| --- | --- | --- | --- | --- | --- | --- | --- | --- | --- |
|  | **1**, N = 798^1^ | **0**, N = 95^1^ | **p-value**^2^ | **1**, N = 342^1^ | **0**, N = 40^1^ | **p-value**^3^ | **1**, N = 244^1^ | **0**, N = 19^1^ | **p-value**^3^ |
| **Sex** |  |  | 0.777 |  |  | 0.865 |  |  | 0.005 |
| Female | 509 (64%) | 62 (65%) |  | 209 (61%) | 25 (63%) |  | 153 (63%) | 18 (95%) |  |
| Male | 289 (36%) | 33 (35%) |  | 133 (39%) | 15 (38%) |  | 91 (37%) | 1 (5%) |  |
| **Age** |  |  | <0.001 |  |  | 0.026 |  |  | 0.033 |
| Mean ± SD | 54 ± 12 | 60 ± 13 |  | 54 ± 12 | 59 ± 12 |  | 54 ± 12 | 62 ± 16 |  |
| **Location** |  |  | 0.040 |  |  | 0.277 |  |  | 0.297 |
| LLL | 148 (19%) | 28 (29%) |  | 61 (18%) | 13 (33%) |  | 31 (13%) | 3 (16%) |  |
| LUL | 267 (33%) | 35 (37%) |  | 104 (30%) | 9 (23%) |  | 85 (35%) | 11 (58%) |  |
| RLL | 84 (11%) | 9 (9%) |  | 38 (11%) | 5 (13%) |  | 27 (11%) | 1 (5%) |  |
| RML | 48 (6%) | 5 (5%) |  | 30 (9%) | 2 (5%) |  | 19 (8%) | 0 (0%) |  |
| RUL | 251 (31%) | 18 (19%) |  | 109 (32%) | 11 (28%) |  | 82 (34%) | 4 (21%) |  |
| **Mean_ct_value** |  |  | 0.987 |  |  | 0.698 |  |  | 0.357 |
| Mean ± SD | -621 ± 145 | -621 ± 197 |  | -619 ± 135 | -606 ± 198 |  | -611 ± 130 | -550 ± 278 |  |
| **Maximum_ct_value** |  |  | 0.683 |  |  | 0.574 |  |  | 0.171 |
| Mean ± SD | -187 ± 298 | -172 ± 362 |  | -181 ± 289 | -150 ± 337 |  | -75 ± 293 | -195 ± 358 |  |
| **Minimum_ct_value** |  |  | 0.652 |  |  | 0.321 |  |  | 0.013 |
| Mean ± SD | -901 ± 131 | -895 ± 142 |  | -898 ± 151 | -916 ± 102 |  | -939 ± 88 | -830 ± 172 |  |
| **Median_ct_value** |  |  | 0.969 |  |  | 0.710 |  |  | 0.277 |
| Mean ± SD | -632 ± 149 | -631 ± 200 |  | -632 ± 130 | -621 ± 188 |  | -627 ± 126 | -554 ± 283 |  |
| **standard_deviation** |  |  | 0.201 |  |  | 0.415 |  |  | 0.005 |
| Mean ± SD | 134 ± 49 | 127 ± 48 |  | 134 ± 47 | 129 ± 41 |  | 146 ± 46 | 118 ± 37 |  |
| **Kurtosis** |  |  | 0.076 |  |  | 0.088 |  |  | 0.340 |
| Mean ± SD | 0.60 ± 1.61 | 0.88 ± 1.45 |  | 0.59 ± 1.45 | 1.31 ± 2.58 |  | 0.89 ± 1.76 | 0.62 ± 1.14 |  |
| **Skewness** |  |  | 0.849 |  |  | 0.377 |  |  | 0.015 |
| Mean ± SD | 0.51 ± 0.51 | 0.50 ± 0.60 |  | 0.77 ± 4.43 | 0.53 ± 0.76 |  | 0.59 ± 0.51 | 0.22 ± 0.59 |  |
| **Entropy** |  |  | 0.281 |  |  | 0.132 |  |  | <0.001 |
| Mean ± SD | 4.88 ± 1.25 | 5.05 ± 1.49 |  | 4.93 ± 1.28 | 5.31 ± 1.50 |  | 5.64 ± 1.25 | 4.66 ± 0.91 |  |
| **Compactness** |  |  | 0.094 |  |  | 0.667 |  |  | 0.681 |
| Mean ± SD | 0.58 ± 0.08 | 0.56 ± 0.06 |  | 0.58 ± 0.08 | 0.58 ± 0.09 |  | 0.57 ± 0.08 | 0.57 ± 0.06 |  |
| **sphere** |  |  | 0.169 |  |  | 0.676 |  |  | 0.604 |
| Mean ± SD | 0.83 ± 0.04 | 0.83 ± 0.03 |  | 0.83 ± 0.04 | 0.83 ± 0.05 |  | 0.83 ± 0.04 | 0.83 ± 0.03 |  |
| **Energy×107** |  |  | 0.397 |  |  | 0.226 |  |  | 0.023 |
| Mean ± SD | 4.61 ± 18.89 | 3.90 ± 4.93 |  | 3.75 ± 11.52 | 12.08 ± 42.63 |  | 3.70 ± 2.48 | 5.38 ± 2.91 |  |
| **Surface** |  |  | 0.079 |  |  | 0.125 |  |  | <0.001 |
| Mean ± SD | 35 ± 54 | 54 ± 100 |  | 35 ± 54 | 63 ± 110 |  | 61 ± 87 | 21 ± 10 |  |
| **Maximum3Ddiameter** |  |  | 0.120 |  |  | 0.107 |  |  | <0.001 |
| Mean ± SD | 6.5 ± 3.7 | 7.5 ± 5.8 |  | 6.5 ± 3.6 | 8.2 ± 6.2 |  | 8.7 ± 5.1 | 5.7 ± 1.6 |  |
| **Costopleura.distance** |  |  | 0.049 |  |  | 0.504 |  |  | 0.114 |
| Mean ± SD | 11 ± 8 | 9 ± 8 |  | 11 ± 8 | 10 ± 7 |  | 10 ± 6 | 6 ± 9 |  |
| **Mass** |  |  | 0.058 |  |  | 0.075 |  |  | <0.001 |
| Mean ± SD | 93 ± 284 | 195 ± 511 |  | 93 ± 270 | 282 ± 647 |  | 205 ± 468 | 48 ± 64 |  |
| **Volume** |  |  | 0.020 |  |  | 0.071 |  |  | <0.001 |
| Mean ± SD | 118 ± 427 | 284 ± 669 |  | 125 ± 305 | 360 ± 794 |  | 535 ± 1,232 | 91 ± 66 |  |
| ^1^n (%) | | | | | | | | | |
| ^2^Pearson's Chi-squared test; Welch Two Sample t-test | | | | | | | | | |
| ^3^Fisher's exact test  1,Early-Stage Tumors;0,Benign Lesions. | | | | | | | | | |
